# Nitrogen removal in freshwater sediments of riparian zone: N-loss pathways and environmental controls

**DOI:** 10.3389/fmicb.2023.1239055

**Published:** 2023-08-17

**Authors:** Fei Ye, Lei Duan, Yaqiao Sun, Fan Yang, Rui Liu, Fan Gao, Yike Wang, Yirong Xu

**Affiliations:** ^1^School of Water and Environment, Chang’an University, Xi’an, China; ^2^Key Laboratory of Subsurface Hydrology and Ecological Effects in Arid Region, Ministry of Education, Chang’an University, Xi’an, China; ^3^Power China Northwest Engineering Corporation Limited, Xi’an, Shaanxi, China; ^4^Shaanxi Union Research Center of University and Enterprise for River and Lake Ecosystems Protection and Restoration, Xi’an, Shaanxi, China

**Keywords:** riparian zone, denitrification, nitrogen removal, stable isotope technology, functional genes

## Abstract

The riparian zone is an important location of nitrogen removal in the terrestrial and aquatic ecosystems. Many studies have focused on the nitrogen removal efficiency and one or two nitrogen removal processes in the riparian zone, and less attention has been paid to the interaction of different nitrogen transformation processes and the impact of *in situ* environmental conditions. The molecular biotechnology, microcosm culture experiments and ^15^N stable isotope tracing techniques were used in this research at the riparian zone in Weinan section of the Wei River, to reveal the nitrogen removal mechanism of riparian zone with multi-layer lithologic structure. The results showed that the nitrogen removal rate in the riparian zone was 4.14–35.19 μmol·N·kg^−1^·h^−1^. Denitrification, dissimilatory reduction to ammonium (DNRA) and anaerobic ammonium oxidation (anammox) jointly achieved the natural attenuation process of nitrogen in the riparian zone, and denitrification was the dominant process (accounting for 59.6%). High dissolved organic nitrogen and nitrate ratio (DOC:NO_3_^−^) would promote denitrification, but when the NO_3_^−^ content was less than 0.06 mg/kg, DNRA would occur in preference to denitrification. Furthermore, the abundances of functional genes (*norB, nirS, nrfA*) and anammox bacterial 16S rRNA gene showed similar distribution patterns with the corresponding nitrogen transformation rates. Sedimentary NO_X_^−^, Fe(II), dissolved organic carbon (DOC) and the nitrogen transformation functional microbial abundance were the main factors affecting nitrogen removal in the riparian zone. Fe (II) promoted NO_3_^−^ attenuation through nitrate dependent ferrous oxidation process under microbial mediation, and DOC promotes NO_3_^−^ attenuation through enhancing DNRA effect. The results of this study can be used for the management of the riparian zone and the prevention and control of global nitrogen pollution.

## 1. Introduction

As a basic component of organisms, nitrogen is also a necessary nutrient element for biological growth (Liu et al., 2019). According to statistics, with the growth of the world’s population and the intensification of agricultural production methods (Basu et al., 2022), the annual amount of nitrogen produced by human activities (about 210 Tg) is 3.6 times that of biological natural nitrogen fixation (Fowler et al., 2013; Zheng et al., 2021). A large amount of anthropogenic active nitrogen is input into terrestrial and marine river systems (Kuypers et al., 2018), which will lead to environmental problems such as eutrophication and acid rain worldwide (Lyu et al., 2021). Although a series of river protection policies have been implemented from local to international (Basu et al., 2022), the nitrate (NO_3_^−^) load in the river basin is still high and even increasing (Wurtsbaugh et al., 2019), which would affect water quality and environmental sustainability (Hu et al., 2023). At the same time, excessive NO_3_^−^/NO_2_^−^ will interact with hemoglobin to generate methemoglobin, which will lose the ability to transport oxygen (Canfield et al., 2010) and seriously threaten human health. Therefore, the exploration of the biogeochemical processes and removal mechanisms of excess reactive nitrogen (especially NO_3_^−^) in rivers has become the focus of attention in the fields of hydrology and environmental ecology.

Although there have been a lot of comments on the research of nitrogen removal (Sharma and Ahlert, 1977; Bo and Tage, 2002; Lee et al., 2009; Deng et al., 2015; Ro et al., 2018; Sun et al., 2021a), the current understanding of the mechanism of nitrogen removal is far less than the understanding of its production mechanism (Zhao et al., 2021). Previous studies have found that denitrification and anaerobic ammonium oxidation (anammox) can permanently remove NO_3_^−^/NO_2_^−^ in the system in the form of N_2_ under anaerobic or anoxic conditions (Mulder et al., 1995; Hamersley et al., 2009; Zheng et al., 2021). The dissimilatory nitrate reduction to ammonium (DNRA) reaction, as a further NO_3_^−^ reduction process, also plays an important role in controlling nitrate conversion (Deng et al., 2015). Different from denitrification and anammox denitrification, DNRA cannot permanently remove nitrogen in the form of N_2_, but retains the converted nitrogen in the form of ammonia nitrogen (NH_4_^+^-N) in the system under anaerobic/anoxic conditions for biological utilization to promote the nitrogen cycle (Broman et al., 2021; Chen et al., 2022). For example, NH_4_^+^ can be oxidized to N_2_ by anammox bacteria (Hu et al., 2019), and can also be converted to NO_3_^−^ by nitrification driven by ammonia-oxidizing archaea, bacteria and nitrite-oxidizing bacteria to provide substrates for denitrification (Wang et al., 2019).

Recent reports showed that denitrification and DNRA processes also occurred under aerobic conditions (Huang et al., 2020; Wen et al., 2020), and they were two microbial processes that compete for NO_3_^−^ and dissolved organic carbon (DOC) (Jia et al., 2020), and the dominant position of the two processes was directly affected by environmental conditions. DNRA occurs preferentially at high carbon load (DOC: NO_3_^−^ > 10) (Chen et al., 2022). The competition and symbiosis mechanism of different nitrogen transformation processes reflects the complexity of the nitrogen cycle process. In addition, some reports have shown that the nitrogen transformation process is controlled by microbial community structure, dissolved oxygen (DO), pH, DOC, iron and nitride content (Ding et al., 2017; Liu et al., 2017, 2022). Among them, DOC is considered to be a key factor limiting nitrogen conversion (Niswonger et al., 2017; Wang L. et al., 2022), and iron is considered to be an important element affecting the removal of nitrate, nitrite and ammonium (Lu et al., 2017; Jamieson et al., 2018). However, the dominant factors under different environmental conditions are different (Chen et al., 2023). Therefore, it is of great significance to fully reveal the mechanism of nitrogen removal and the corresponding environmental control to solve the nitrogen pollution in the basin.

As a transitional zone between river and terrestrial systems, the riparian zone of the river has a significant diversity of species and environmental conditions. It plays an important role in improving water quality and maintaining the health and sustainable development of watershed ecosystems (Zhao et al., 2019; Lyu et al., 2021). It is also considered to be a hot spot area for nitrogen buffering (Wang et al., 2019), which can prevent (remove) about 70% of nitrogen from entering the river (Mayer et al., 2007; Ding et al., 2017). The structural characteristics of sediments restrict the migration and transformation of solutes (Hou et al., 2017; Peralta-Maraver et al., 2018). For example, sediment particles with small particle size will reduce the solute exchange flux by 75% and hinder solute transport. Sediments with coarse particle size can promote solute transport and accelerate microbial reaction rate (Fetzer et al., 2017; Wang et al., 2020b). For the riparian zone with obvious heterogeneity of sediment structure, there are significant differences in nitrogen conversion rate. In addition, the fluctuation of river water and groundwater level controls the redox conditions and hydro-chemical indexes (such as pH, DOC, water content, etc.) of riparian sediments, which further affects the structure of microbial community and N-loss rate (Covatti and Grischek, 2021; Wang et al., 2021). Previous studies on nitrogen transformation in riparian zones showed that denitrification, anammox and DNRA processes were the main nitrogen removal pathways in riparian zones (Li J. et al., 2020; Ruan et al., 2020; Zhao et al., 2021). However, due to the high heterogeneity of riparian zones, the complexity of nitrogen transformation processes and the limitations of research methods and technologies, the contribution and limiting factors of these processes to nitrogen removal in riparian zones are still rarely reported.

In view of this background, this study aims to investigate the habitat conditions, N-loss process and distribution characteristics of related microbial communities under heterogeneous sedimentary structure of riparian zones by molecular biology technology, isotope tracer technology and microcosm control experiment. The purpose of this study is (1) to identify the nitrogen removal process under heterogeneous conditions in riparian zones, (2) to quantify the nitrogen removal rate of the riparian zone and the contribution of main nitrogen removal processes to nitrogen removal, and (3) to reveal the main controlling factors affecting the nitrogen removal in the riparian zone. This study can provide new insights into the nitrogen removal mechanism of riparian zones and help to more accurately assess the purification capacity of riparian zones.

## 2. Materials and methods

### 2.1. Study area and data collection

Located on the Loess Plateau in Northwest China, the Wei River is the largest tributary of the Yellow River, with a total length of 818 km, flowing through Gansu and Shaanxi provinces (Su et al., 2019). The Shaanxi section of the Wei River is 502 km long, which provides water for 60% of the population and 56% of the cultivated land in Shaanxi Province (He et al., 2020). The water flow in this section is from west to east, and the lithology of the riparian sediment gradually becomes finer from the upstream Baoji section (pebbles, gravels) to the downstream Weinan section (fine sand, silt), and the flow rate becomes slower. Affected by the input of excessive nitrogen fertilizer, inorganic nitrogen load (mainly NO_3_^−^) has become the main source of non-point source pollution in the Shaanxi section of the Wei River, and the nitrogen pollution problem in the downstream Weinan section is more serious (Wang et al., 2018; Shi et al., 2019).

**Figure 1:**
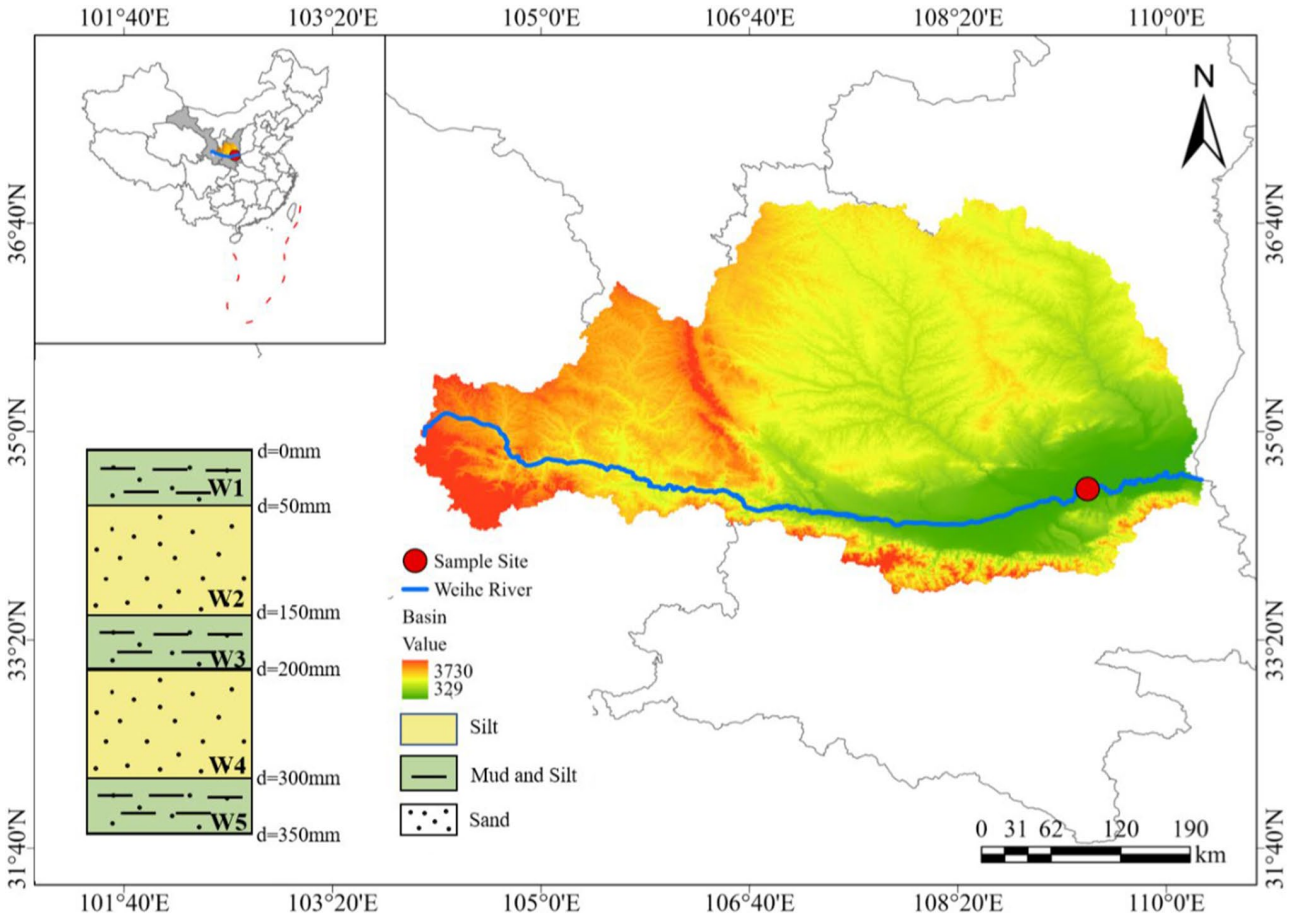
Study area map and distribution of sampling sites.

The riparian zone of the Weinan section was selected as a typical heterogeneous profile and sampling points were set up (shown in Figure 1). According to the natural stratification of the riparian zone profile, the sediment samples of different layers were collected using a portable sediment sampler (Rigo Co., Ltd., Saitama, Japan) after disinfection with anhydrous alcohol. One group of samples (about 10 kg) was collected from each layer, and a total of 5 groups of samples were collected. The samples numbered from top to bottom were W1 (0–50 mm), W2 (50–150 mm), W3 (150–200 mm), W4 (200–300 mm), and W5 (300–350 mm). Among them, W1, W3 and W5 layers were mixed with black sludge. In order to avoid pollution during storage and transportation, the collected samples were packed into sterile self-sealing bags and sterile tube tubes (50 mL), respectively. The samples of sterile self-sealing bags were used for experiments and physical and chemical properties analysis, and were stored at −4°C. The samples of sterile tube tubes were used to determine microbial diversity and abundance, and were stored in an ultra-low temperature refrigerator at −80°C.

### 2.2. Analysis of sediment characteristics

As the sediment samples were collected, the pH value of the sediment was immediately measured using a pH400 portable soil pH meter (Spectrum Technologies, United States), the conductivity and total dissolved solids of the sediment were measured using a 2265FS conductivity meter (Spectrum Technologies, United States), and the water content (WC) and redox potential (ORP) of the sediment were measured using a soil multi-parameter tester (Delta-T WET-2, United States). In addition, some sediment samples were taken from each group to remove debris such as trunks, and then dried naturally and screened by a 2.0 mm filter. The treated samples were analyzed by laser particle size analyzer Mastersizer2000 (Malvern Instruments Ltd., United Kingdom), and the mineral structure of the samples was analyzed by SEM scanning electron microscopy (Thermo Fisher QUANTA 250PEG, United States) (Zhou et al., 2016). The dissolved organic carbon (DOC) in sediments was determined by total organic carbon analyzer (Shimadzu TOC-VCPH, Japan), and the dissolved inorganic carbon (DIC) was measured using a dissolved inorganic carbon analyzer (ApolloAS-C3, United States). The Fe(II) was determined by phenanthroline spectrophotometry (Zheng et al., 2020). After extraction with 2 mol L^−1^ KCl, the contents of total nitrogen (TN), ammonia nitrogen (NH_4_^+^-N), nitrate nitrogen (NO_3_^−^-N) and nitrite nitrogen (NO_2_^−^-N) in sediments were determined by UV-1800 ultraviolet spectrophotometer (Shimadzu, Kyoto, Japan), with detection limits of 0.015, 0.015, and 0.03 mg/kg, respectively. Dissolved organic nitrogen (DON) was determined by continuous flow analyzer (Skalar SAN++, Netherlands). The sample test was carried out in parallel for three times. The test results were expressed as the mean of three tests, and the error range was less than 5%.

### 2.3. DNA extraction and sequencing analysis

After thawing and mixing the sediments at each sampling point, 0.25 g was weighed, and then DNA was extracted in strict accordance with the instructions of PowerSoil ^®^ DNA separation kit (Mo Bio Carlsbad, United States). The concentration and purity of DNA were detected by NanoDrop2000 spectrophotometer (Thermo Scientific, United States), and the qualified DNA samples were analyzed by fluorescence quantitative (qPCR). Sequencing was performed using the Illumina Miseq platform (Personal Biotechnology Company, Shanghai, China). The abundance of genes (*nirS, norB, Amx-16S, nrfA*) related to nitrogen removal processes (i.e., denitrification, anammox and DNRA) in sediment samples was determined by SYBR-Green dye using ABI7500 rapid real-time fluorescence quantitative PCR instrument (Applied Biosystems, Foster City, CA, United States). The Table 1 provides detailed information on primers, and three qPCR analyses were performed for each sample for error analysis.

**Table 1:** Standard primer parameters for qPCR analysis of target genes.

Type	Primer	Target gene	Sequence	References
Denitrify	cd3aF	*nirS*	GTCAACGTGAAGGAAACCGG GAGTTCGGATGGGTCTTGA	Throback et al. (2004)
	R3cdR			
	cnorB2F	*norB*	GACAAGNNNTACTGGTGGT	Braker and Tiedje (2003)
	cnorB6R		GAANCCCCANACNCCNGC	
DNRA	nrfAF1aw	*nrfA*	CARTGYCAYGTSGARTA	Welsh et al. (2014)
	nrfAR1		TWNGGCATRTGRCARTC	Mohan et al. (2004)
Anammox	Amx368f	*16S rRNA*	TTCGCAATGCCCGAAAGG	Schmid et al. (2007)
	Amx820r		AAAACCCCTCTACTTAGTGCCC	

### 2.4. Isotope tracer experiment

In order to quantitatively identify the nitrogen removal process in riparian zones, ^15^N isotope tracing experiments were carried out using isotope pairing technique (IPT) (Nielsen, 1992). The sediment and sterile water were mixed at a mass ratio of 1: 7 to make a uniform mud and filled with helium for 30 min, and then the mud was filled into a series of airtight serum bottles with syringes (Exetainer, Labco, United Kingdom). The bottles were placed in a dark environment for 24 h, and the remaining NO_3_^−^-N and NO_2_^−^-N in the bottles and DO in deionized water were consumed. 3 serum bottles were randomly selected after culture, 200 μL 50% ZnCl_2_ solution was added to inhibit microbial activity, and the remaining NO_3_^−^-N and NO_2_^−^-N concentrations in the bottles were extracted and determined. The remaining serum bottles were divided into 4 groups, numbered E1, E2, E3 and E4. The 100 μL helium-aerated isotope reagents were injected into the first three groups with syringes: E1 (^15^NH_4_^+^ standard solution with 99.6% ^15^N), E2 (^15^NH_4_^+^+^14^NO_3_^−^ standard solution, at a volume ratio of 1,1) and E3 (^15^NO_3_^−^ standard solution with 99% ^15^N) (Zhang et al., 2020), and the final concentration of standard solution in each group was 100 μmol/L. Then, 200 μL of 50% ZnCl_2_ solution was injected into half of the bottles in each group as the initial sample, and 200 μL of 50% ZnCl_2_ solution was injected into the remaining serum bottles after 8 h of culture as the termination sample. The concentration of dissolved N2 (^28^N_2_, ^29^N_2_, ^30^N_2_) in the supernatant was determined by MAT253 stable isotope ratio mass spectrometer (Thermo Fisher Scientifie Inc., MA, United States) to calculate the denitrification and anammox rates.

The E4 was used to determine the DNRA reaction rate, and the injection solution was K^15^NO_3_ (99.0 atom%, final concentration approximately 100 μmol / L) aerated by helium, in addition, the starting and ending samples were filled with high-purity helium for aeration for 30 min to remove N_2_ (^29^N_2_, ^30^N_2_) produced by denitrification and anammox in the bottle (Deng et al., 2015). Then 200 μL HBrO was injected into the aerated serum bottle to completely oxidize ^15^NH_4_^+^ to ^29^N_2_ and ^30^N_2_, and the concentration of N_2_ and ^15^NH_4_^+^ in E4 was determined by mass spectrometer and ultraviolet spectrophotometer (Soonmo and Wayne, 2002; Hou et al., 2013; Yin et al., 2014), all samples needed three parallel experiments. Denitrification (D_total_), anammox (A_total_) and dissimilatory ammonium reduction (R_DNRA_) rates (μmol·N·kg^−1^·h^−1^) were calculated from the following equations (Nils Risgaard-Petersen et al., 2003; Trimmer et al., 2006):


(1) $$D_{total}=Q_{30}\times F_{N}^{-2}$$

(2) $$A_{total}=\frac{Q_{29}+2\times \left(1-F_{N}^{-1}\right)\times Q_{30}}{F_{N}}$$

(3) $$R_{DNRA}=\left({\left[N15H_{4}^{+}\right]}_{2}-\left[N15H_{4}^{+}\right]1\right)\times W^{-1}\times T^{-1}$$

Where Q_30_ is the formation rate of ^30^N_2_ measured by mass spectrometer, F_N_ is the proportion of ^15^NO_3_^−^ in total NO_3_^−^ after adding ^15^NO_3_^−^, Q_29_ is the formation rate of ^29^N_2_ measured by mass spectrometer, [^15^NH_4_^+^]_1_ is the concentration of ^15^NH_4_^+^ in the system at the initial time, [^15^NH_4_^+^]_2_ is the concentration of ^15^NH_4_^+^ μmol/L in the system after 8 h of culture, W is the sample mass kg, and T is the culture time h. Analysis of variance (ANOVA) was used to examine the spatial and temporal differences in denitrification, anammox and DNRA rates.

### 2.5. Microcosm static culture experiment

In order to further explore the role of Fe(II) and DOC in the N-loss process of riparian zone, batch static culture experiments were carried out in anaerobic glove box (COY-7150220, United States) by controlling variables. The samples were evenly mixed with each layer of sediment samples in riparian zone. Anaerobic deionized water (Ar/N_2_ for 20 min) was mixed with sediment samples at a ratio of 3: 1, and 12 g of the above homogeneous mixed slurry was taken into a 100 mL serum bottle (in triplicate). After standing, 80 mL of pure water was added, and then Ar/N_2_ was introduced for aeration for 20 min, and the bottle was filled with anaerobic deionized water. Cultured under anaerobic dark conditions for 2 days to remove the sample background to remove nitrate, nitrite and oxygen from the sample background. Different exogenous substances were added to each bottle (details in Supplementary Tables S1, S2), and anaerobic dark culture was performed for 31 days. The supernatant was collected regularly with a 1 mL syringe (interval of 24 h on the first 15 days, and interval of 48 h on the 16th day) to determine the concentration of Fe(II), nitrate nitrogen, nitrite nitrogen and ammonia nitrogen by the phenanthroline spectrophotometry and UV-1800 ultraviolet spectrophotometer (Shimadzu, Kyoto, Japan), all samples need three parallel experiments. After each sampling, it was sealed with a sealing film to prevent oxygen pollution.

### 2.6. Statistical analysis

In this study, the OriginPro 2022 and R language were used for graphic drawing and analysis. One-way analysis of variance (ANOVA) was used to test the differences in nitrogen conversion rate, microbial functional genes and sediment physical and chemical indicators (95% confidence interval). The SPSS software package (version; 25 IBM, United States) was used to estimate the relationship between different variables through pearson correlation and linear regression equation. The significance level of all statistical analyses was set as *p* < 0.05.

## 3. Results and discussion

### 3.1. Physicochemical properties and spatial distribution characteristics of nitrogen in riparian sediments

The lithology structure of the riparian zone in the study area is heterogeneous in the vertical direction (Table 2). The soil particle size ranged from 1.98 to 215.9 μm, mainly silt (4–63 μm), with clay (>63 μm), silt, and fine sand (<4 μm) contents accounting for 0–0.03%, 72.15–98.1%, and 1.86–27.85%, respectively. There were abundant minerals in the sediments of the riparian zone, mainly quartz (55.9–64.1%) and plagioclase (14.8–20.0%). Followed by calcite (6.0–8.6%), chlorite (4.7–6.6%), illite (3.4–5.4%), potassium feldspar (1.7–4.4%), dolomite (1.3–4.1%) and a small part of amphibole, which indicated that the parent rock of the sediment was completely weathered and the mineral maturity was high.

**Table 2:** Physical and chemical characteristics of sediments at sampling sites.

Sampling site	W1	W2	W3	W4	W5
Depth (mm)	0–50	51–150	151–200	201–300	301–350
Caly (%)	0 ± 0.01	0 ± 0.01	0 ± 0.01	0 ± 0.01	0.03 ± 0.01
Silt (%)	82.14 ± 0.39	74.07 ± 1.12	72.15 ± 0.83	72.37 ± 2.35	98.12 ± 0.64
Fine sand (%)	17.86 ± 0.22	24.93 ± 0.37	27.85 ± 0.28	27.63 ± 0.13	1.86 ± 0.06
pH	7.79 ± 0.12	8.02 ± 0.29	7.77 ± 0.08	7.96 ± 0.32	7.78 ± 0.11
ORP (mV)	144.31 ± 12.89	130.21 ± 14.26	80.53 ± 9.31	40.28 ± 5.67	23.53 ± 9.89
EC (μS/cm)	535.17 ± 15.22	248.31 ± 10.35	307.46 ± 10.45	209.28 ± 12.61	257.75 ± 11.43
TDS (mg/L)	267.34 ± 11.47	124.22 ± 11.19	153.47 ± 12.28	104.42 ± 9.13	128.54 ± 8.89
Water content (%)	26.12 ± 0.89	22.38 ± 1.21	25.16 ± 0.77	24.85 ± 1.16	23.87 ± 0.78
Fe(II) (mg/kg)	39.84 ± 3.22	42.72 ± 2.34	98.65 ± 1.22	103.15 ± 3.9	252.33 ± 5.21
DOC (mg/kg)	76.23 ± 9.23	41.06 ± 7.89	70.57 ± 8.56	54.23 ± 7.88	68.57 ± 9.91
DIC (mg/kg)	6.84 ± 1.34	3.84 ± 0.72	5.18 ± 0.67	5.07 ± 1.45	4.95 ± 1.55

ORP is redox potential, EC is conductivity, and TDS is total dissolved solids, and numbers after ± represent standard deviations (*n* = 3).

The soil particles of the riparian zone sediments had a high degree of polymerization, and micro-aggregates were observed in the sediment W1, W3, and W5 layers containing sludge, and there were obvious bridging substances between the soil particles (Supplementary Figure S1), which greatly hindered the uptake of organic matter by microorganisms, protected the ‘comprehensiveness’ of soil organic matter, and formed natural organic carbon. Therefore, the DOC content in this area was high (Table 2). In addition, the pH range of the sediment environment in the riparian zone was 7.77–8.02, which was weakly alkaline overall. Along the vertical direction of the riparian zone, conductivity (EC) and total dissolved solids (TDS) showed a decreasing trend, Fe(II) gradually increased, and oxidation reduction potential (ORP) gradually decreased, and the reducibility of the sedimentary environment in the riparian zone gradually increased.

**Figure 2:**
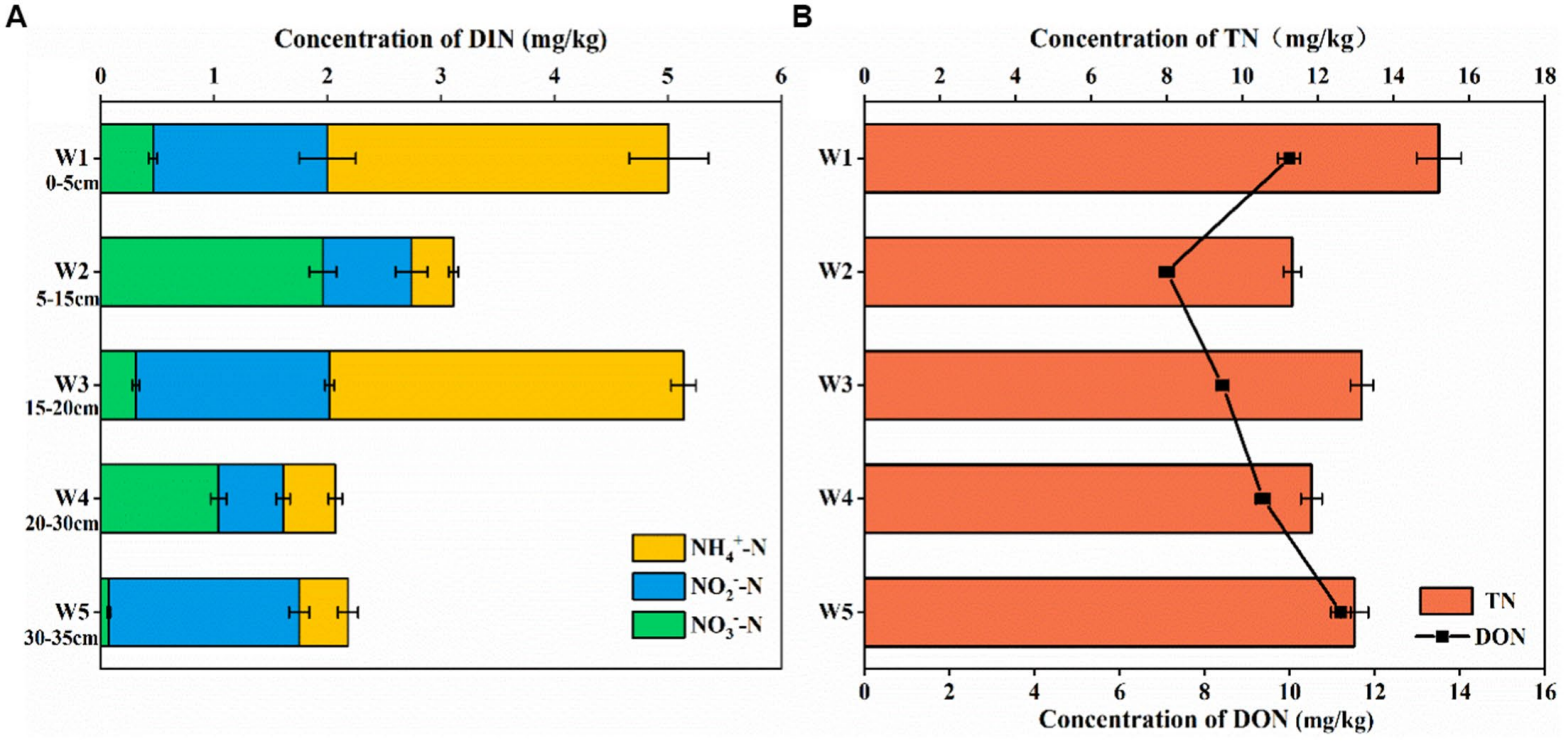
Vertical distribution of nitrogen in riparian sediments of Weinan section of Wei River [**(A)** The content of DIN, **(B)** The content of TN and DON, Error bars represent standard deviations].

As shown in Figure 2, the distribution of nitrogen content in different depths of sediments in the riparian zone of the Weinan section of the Wei River varied greatly, with the total nitrogen (TN) content ranged from 11.8 to 15.31 mg/kg, and the DON content ranged from 7.21 to 11.42 mg/kg, which was the main form of nitrogen. With the increase of sediment depth in the riparian zone, TN showed a downward trend, DON showed an increasing trend (Figure 2A), and dissolved organic nitrogen (DIN) content gradually decreased (Figure 2A), which confirmed that the riparian zone had a removal effect on inorganic nitrogen pollution.

It had been shown that the migration and transformation of nitrogen in the riparian zone is mainly completed by the geochemical biological effects of NH_4_^+^-N, NO_3_^−^-N and NO_2_^−^-N (nitrification, denitrification, anaerobic ammonium oxidation, etc.) (Zhao et al., 2021). In the riparian sediments of Weinan section of Wei River, the NO_3_^−^-N, NO_2_^−^-N and NH_4_^+^-N contents were 0.06–1.96 mg/kg, 0.03–1.71 mg/kg and 0.42–3.12 mg/kg, respectively. In layers W1, W3, and W5, which contained sludge, the DIN in the sediments mainly existed in the form of NO_2_^−^-N and NH_4_^+^-N, and the NO_3_^−^-N content was low and reached a minimum value of 0.06 mg/kg in the lowest layer W5, while in the sediment layers W2 and W4, the content of NO_3_^−^-N was significantly higher than that of NO_2_^−^-N and NH_4_^+^-N, which indicated that there was a complex nitrogen cycle in the sediments of the riparian zone in Weinan section.

### 3.2. Microbial community dynamics involved in nitrogen removal processes in riparian sediments

**Figure 3:**
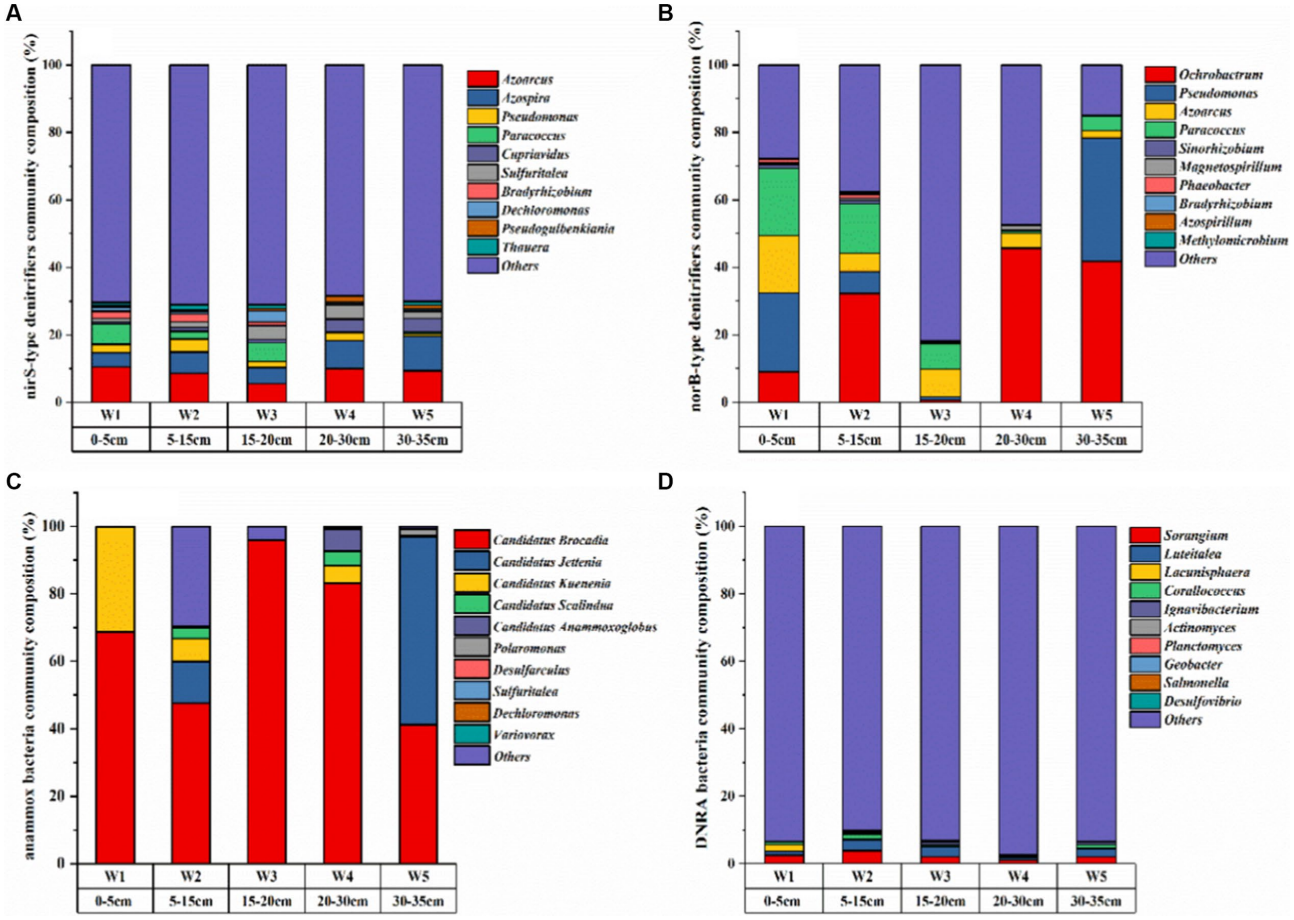
The community composition of nitrogen-transforming microorganisms in the riparian sediments of the Weinan section of the Wei River [top 10 species at the sample genus level: **(A)** *nirS* denitrifying bacteria; **(B)** *norB* denitrifying bacteria; **(C)** anammox bacteria; **(D)** DNRA bacteria].

Metagenomics can determine the composition and function of microbial communities (Daims et al., 2015; Chen et al., 2022). In this study, genes associated with denitrification (*nirS, norB*), anammox (Amx-16S) and DNRA (nrfA) reactions were detected in the sediments of the riparian zone of the Weinan section of the Wei River by qPCR, which indicated that the riparian zone may remove nitrogen from the environment through denitrification, anammox and DNRA processes. Among them, denitrification combined with anammox removed NO_3_^−^-N ‘permanently’ from the riparian zone of the Wei River, while DNRA converted NO_3_^−^-N into inorganic nitrogen and retained it in the riparian zone for other organisms (Crowe et al., 2012; Deng et al., 2015). The Coverage index of each sample in this study was greater than 0.99, indicating that the determination results of sediment samples had high confidence (Supplementary Table S3). There were significant differences in the diversity and abundance of microbial communities involved in the nitrogen removal process along the sediment depth (Figure 3).

The diversity of denitrifying bacteria community in the riparian zone of Weinan section was the highest, and *Proteobacteria* was the most important denitrifying bacteria (Figures 3A,B), which was the same as the results of other freshwater system sediments (Song et al., 2012; Dong et al., 2021). Aerobic denitrifying bacteria such as *Azoarcus, Ochrobactrum, Pseudomonas*, *Paracoccus*, and *Azospira* are the main dominant genera (Figures 3A,B). Which was attributed to the interaction between river water and groundwater. The transport of dissolved oxygen to sediments provided an oxidizing environment for microorganisms. The high redox potential in sediments also confirmed this view (Table 2). These aerobic microorganisms use oxygen/NO_3_^−^-N/NO_2_^−^-N as an electron acceptor for denitrification under aerobic conditions (Huang et al., 2021; Zhang et al., 2022).

**Figure 4:**
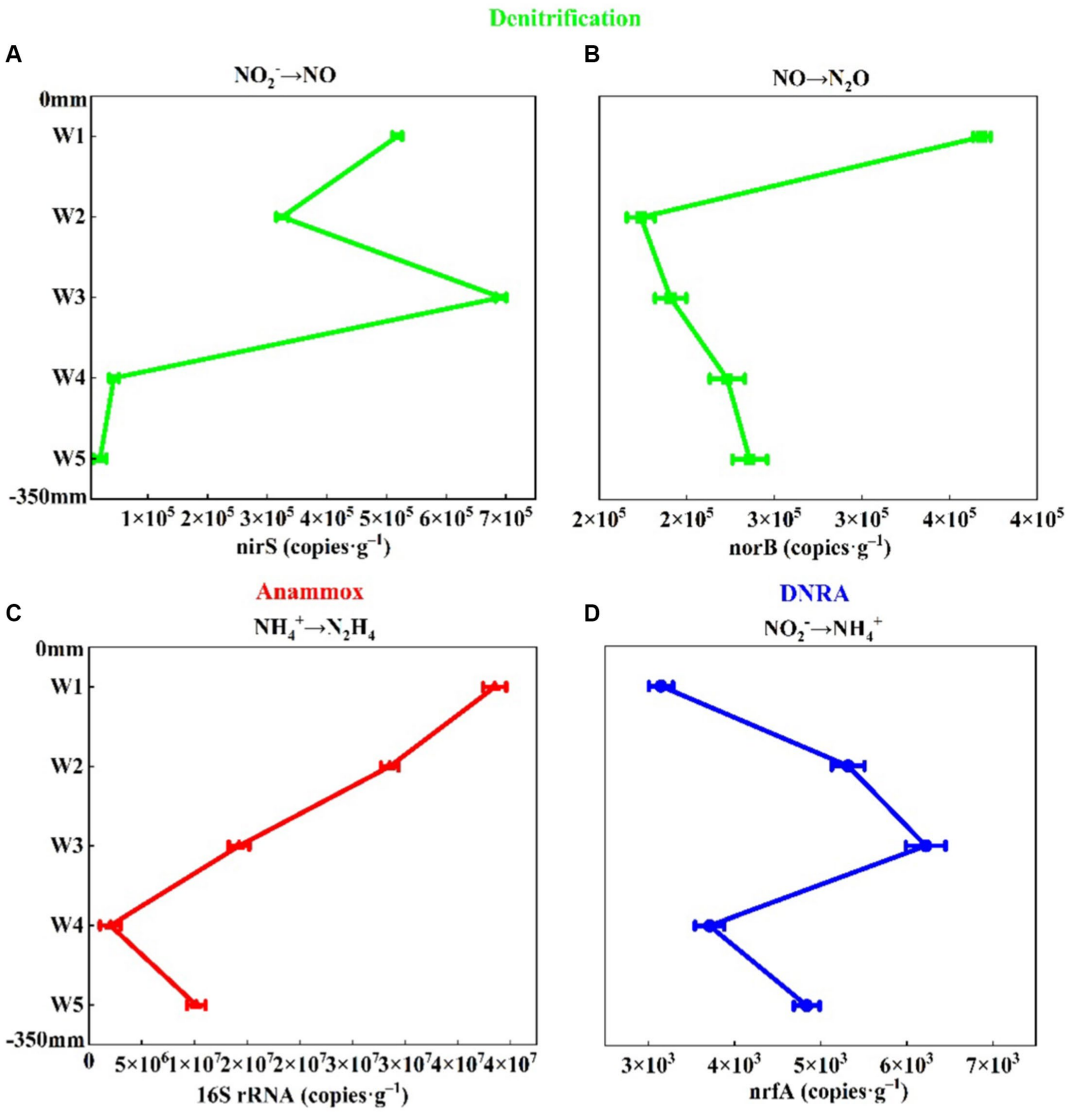
Abundance of genes for nitrogen removal in riparian sediments of Weinan section of Wei River [**(A)** nirS, **(B)** norB, **(C)** 16S rRNA, **(D)** nrfA, Error bars represent standard deviations].

**Figure 5:**
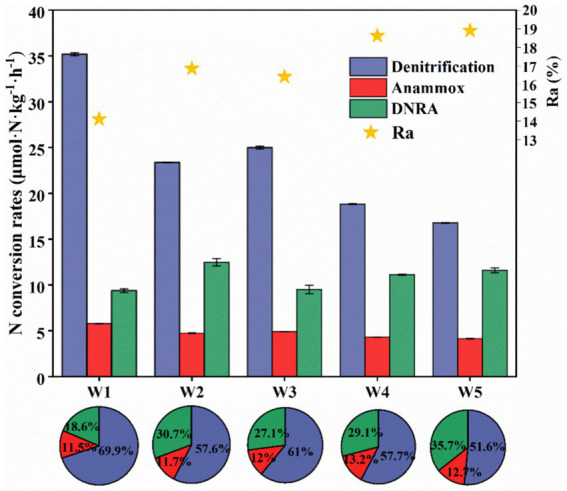
The spatial variation characteristics of denitrification, anammox and DRNA rates in the riparian zone of Weinan section of Weihe River (Ra is the N_2_ contribution of anammox rate in the N-loss process); the pie chart is the contribution of potential nitrogen removal rate, anammox rate and DNRA rate to the attenuation of NO_3_^−^-N or NO_2_^−^-N in different depth samples of riparian zone in the study area, Error bars represent standard deviations.

In addition, the abundance of *nirS* gene was 2.01 × 10^4^–6.92 × 10^5^ copies g^−1^, and the abundance of *norB* gene was 1.74 × 10^5^–3.68 × 10^5^ copies g^−1^ (Figure 4). As a nitrite reductase marker gene *nirS* (Wang et al., 2019), the abundance of *nirS* gene was the same as the distribution of NO_2_^−^-N content (Figures 2A, 4A). With the increase of sediment depth, the abundance of *nirS* gene decreased first and then increased, and then decreased gradually, and decreased to the minimum in W5 layer (Figure 4A). As a marker gene of nitric oxide reductase (Liu et al., 2017), the abundance of *norB* gene was similar to the distribution of N_2_O content (Figure 4B and Supplementary Figure S2). The abundance of *norB* gene decreased sharply from W1 layer, and decreased to the minimum value in W2 layer and then increased gradually. It was shown that sediment denitrification capacity was positively correlated with the abundance of the *nirS* gene (Mosier and Francis, 2010). We also found that the spatial distribution pattern of *nirS* gene abundance and denitrification rate in the riparian zone of Weinan section was the same (Figures 4A, 5), which was higher in W1 and W3 layers and lower in other layers, which further indicated that the abundance of *nirS* gene could be approximated as an indicator of denitrification capacity in sediments.

The dominant genera of anammox bacteria in the riparian zone of Weinan section of Wei River were *Candidatus Brocadia*, *Candidatus Jettenia*, *Candidatus Kuenenia* and *Candidatus Scalindua* (Figure 3C). Most of these dominant bacteria were autotrophic, which used inorganic carbon as a carbon source in the anaerobic environment, and were anaerobic ammonia-oxidizing bacteria commonly found in terrestrial ecosystems (Humbert et al., 2010). The lowest diversity and relatively homogeneous composition of anammox bacteria were found in the sludgy layers W1, W3 and W5 (Figure 3C), which could be attributed to the fact that the high DOC content in the sludgy layer inhibited the growth of anammox bacteria (Guven et al., 2005; Chamchoi et al., 2008). The abundance of anammox bacteria 16S rRNA gene was the highest in riparian sediments, which was two orders of magnitude higher than the abundance of denitrifying functional genes, ranging from 2.03 × 10^6^ to 3.85 × 10^7^ copies g^−1^ (Figure 4C), and gradually decreased along the sediment depth.

The dominant genera of DNRA bacteria in riparian zone were mainly *Sorangium*, *Luteitalea*, *Lacunisphaera* and *Corallococcus* (Figure 3D), which were similar to the reports of DNRA bacterial diversity in other rivers and lakes (Yuan et al., 2022; Zhao et al., 2022), and most of these microorganisms were heterotrophic (Wang et al., 2020a). Among them, *Sorangium* is an aerobic bacterium belonging to *Myxococcales*, which can use NO_3_^−^-N/NO_2_^−^-N to produce NO (Banerjee et al., 2018), and is considered to be a key microorganism for synergistic control of soil microbial composition. *Luteitalea* belongs to *Acidobacteria*, which are aerobic and chemoheterotrophic bacteria, and can use organic matter in the environment as a carbon source for life activities (Vieira et al., 2017; Qiao et al., 2020). Compared with other functional genes, the abundance of *nrfA* gene in DNRA bacteria was the lowest, ranging from 3147.43–6221.28 copies g^−1^ (Figure 4D). Meanwhile, along the depth direction of the sediment, the abundance of *nrfA* gene was completely consistent with the distribution of DOC content (Figure 4D and Table 2). DOC, as an important carbon source of DNRA, can promote DNRA reaction (Broman et al., 2021), which is an important factor in regulating DNRA in riparian zone.

Microorganisms are important mediators for nitrogen migration and transformation in riparian sediments (Kuypers et al., 2018; Liu et al., 2021). Our observations showed that heterogeneous riparian zones can provide microhabitats for denitrifying, anaerobic ammonia oxidation and DNRA bacteria. The differences in the diversity and abundance of these communities in sediment layers with different lithologic structures may affect the nitrogen removal process of the riparian zone.

### 3.3. Nitrogen removal mechanism and nitrogen removal capacity of riparian sediments

As shown in Figure 5, we calculated the denitrification rate (D_total_), anammox rate (A_total_) and DNRA rate (R_DNRA_), which further confirmed that the natural attenuation process of nitrogen in the riparian zone of the study area was achieved by the combined action of these three processes. Unlike other regions where denitrification and anammox reactions are the main N-loss pathways in sediments (Choi et al., 2016; Liu et al., 2019; Zhang et al., 2020; Zheng et al., 2020), the contribution rate of anammox to nitrogen removal in the riparian zone of Weinan section of Wei River was extremely low (about 12.2%), and A_total_ was 4.14–5.77 μmol·N·kg^−1^·h^−1^ (Figure 5), which was related to the unique properties of the riparian zone. Under the interaction of river water and groundwater, the sedimentary environment of the riparian zone was oxidizing and heterogeneous, while anammox bacteria were extremely sensitive and had extremely high requirements for environmental stability, using inorganic carbon (DIC) as a carbon source for anammox reactions in low or even anaerobic environments (Jin et al., 2012; Huang et al., 2022), and the content of DIC in the riparian zone was low (Table 2), thus the activity of anammox bacteria was suppressed. Even though the oxidation of the environment along the sediment depth direction became weaker, the abundance of anammox bacteria also decreased (Figure 4) and A_total_ remained low. However, the anammox process contributed 14–19% of the N_2_ production (Figure 5), and its nitrogen removal capacity cannot be ignored.

In contrast, denitrification and DNRA played a major role in the nitrogen removal process in the riparian zone of Weinan section of Wei River, and their nitrogen removal contribution rates were about 59.6 and 28.2%, respectively. Among them, D_total_ was 17.76–35.19 μmol·N·kg^−1^·h^−1^, R_DNRA_ was 9.37–12.47 μmol·N·kg^−1^·h^−1^ (Figure 5). DNRA and denitrification belong to the dissimilatory reduction process of NO_3_^−^-N, and both of them use NO_3_^−^ as the electron acceptor with substrate competition (Yin et al., 2017; Wang et al., 2020b; Wang Z. et al., 2022). It is generally believed that denitrification requires less free energy than DNRA action in the same environment, and thus preferentially occurs in most cases (Kartal et al., 2007; Chen et al., 2015). Meanwhile, the community diversity and abundance of denitrifying bacteria in riparian zone sediments were much higher than that of DNRA bacteria (Figures 3, 4), thus denitrification dominated the N-loss process in riparian zones (Figure 5).

In addition, we also observed that both D_total_ and R_DNRA_ showed significant spatial distribution characteristics. Along the sediment depth direction, D_total_ decreased first and then increased, and then gradually decreased (Figure 5), which was similar to the variation of denitrifying bacteria (especially *nirS* gene) abundance and its contribution rate to nitrogen removal (Figure 4A). The R_DNRA_ fluctuated slightly, but with the increase of sediment depth, the contribution rate of DNRA to nitrogen removal increased significantly, and reached the maximum value of 35.7% in the bottom layer W5 (Figure 5), which we speculated might be related to the competitive mechanisms of denitrification and DNRA. Studies have shown that DOC: NO_3_^−^ (electron donor: acceptor) is a key factor in regulating denitrification and DNRA (Tiedje et al., 1983; Rütting et al., 2011), but in the sediment of a single medium, it was not DOC: NO_3_^−^ but resource concentration (NO_3_^−^) that determines the choice of DNRA and denitrification pathways, and when the content of NO_3_^−^ becomes more limited, it is more conducive to DNRA (Vuono et al., 2019). Although the sediments in the riparian zone of the Weinan section were heterogeneous, the lithology structure of the bottom W5 layer was relatively simple, and the silt (accounting for about 98%) was the main lithology structure (Table 2). Meanwhile, the minimum NO_3_^−^ content in the W5 layer was only 0.06 mg/kg, which might promote the NO_3_^−^ to select the reduction path of DNRA and increase the contribution rate of DNRA in this layer.

Previous studies have shown that the abundance of functional genes is a good predictor of nitrogen transformation rate (Wang et al., 2017; Zhang et al., 2018). Interestingly, in this study, although the abundance of 16S rRNA gene of anammox bacteria was the highest and the abundance of *nrfA* gene of DNRA bacteria was the lowest, the anammox rate in riparian sediments was significantly lower than that of DNRA (Figure 5). Therefore, the abundance of microbial genes cannot systematically quantify the biogeochemical process of nitrogen. The nitrogen cycle in riparian zone is not only determined by microorganisms, but also related to the nature of riparian zone itself, environmental factors and specific nitrogen transformation process. This study shows that molecular biology techniques coupled with isotope tracing methods can better quantitatively reveal the N-loss process in the riparian zone.

To evaluate the nitrogen removal efficiency of the riparian zone of Weinan section of Wei River, we compared the nitrogen removal rate of sediments from different aquatic ecosystems in various regions of the world (Table 3). We found that the nitrogen removal rate of sediments in the Weinan section of the Wei River was 4.14–35.19 μmol·N·kg^−1^·h^−1^, which was higher than that of sediments in most regions, including wetlands, continental shelf and estuary sediments. This indicates that the riparian zone of Weinan section has a strong capacity to reduce inorganic nitrogen pollution. We also found that the nitrogen removal rate of riparian sediments in the study area was similar to that of estuarine turbidity maximum zone (TMZ) and wetland. In recent years, TMZ has been considered as an important place to remove reactive nitrogen from land to sea due to its large number of suspended particles that can easily adsorb reducing cations to consume oxygen, provide a growth environment for anaerobic microorganisms, and promote denitrification and anammox reactions (DeVries et al., 2012; Zheng et al., 2021), and wetland is considered to be a hot spot for nitrogen removal due to its high organic carbon and anaerobic environment prone to denitrification (Cheng et al., 2020). Therefore, the sediments in the riparian zone of the Weinan section share some common characteristics with TMZ and wetland, such as high organic matter content, low oxygen concentration and diverse microbial communities.

**Table 3:** Comparison of nitrogen removal efficiency of sediments from different aquatic systems.

Location	Method	Nitrogen removal rate μmol·N·kg^−1^·h^−1^	References
Shihwa Swap, South Korea	N stable isotope	0.29–3.86	Ro et al. (2018)
Indus River estuary	N stable isotope	0.01–6.27	Fozia et al. (2020)
Fall Creek Riparian zone, America	N stable isotope	2.09–4.1	McPhillips et al. (2015)
Eastern Coastal Continental Shelf, China	N stable isotope	0.03–6.53	Sun et al. (2021b)
Changjiang Estuary, China	N stable isotope	0.06–4.51	Deng et al. (2015)
TMZ of the Yangtze River Estuary, China	N stable isotope	3.5–35.3	Zheng et al. (2021)
The East Sea, China	N stable isotope	0–36.6	Lin et al. (2017)
Taihu Lake, China	Acetylene reduction	0–0.258	Yao et al. (2018)
Green Bay Wetland, China	Stable isotope	0.78–21.29	Shen et al. (2018)
Weinan Riparian Zone of Weihe River, China	N stable isotope	4.14–35.19	This study

The nitrogen conversion rate in the table is the minimum-maximum value of each nitrogen migration conversion rate involved in the N-loss process.

### 3.4. Effects of Fe (II) and DOC on N–loss process in riparian zone

Iron and DOC are important components in the riparian zone, and they can regulate the nitrogen transformation process in environmental media (Wu et al., 2021; Lu et al., 2022), which plays an important role in the natural attenuation of nitrogen (Yang et al., 2018; Lu et al., 2022). Large amounts of Fe(II) and DOC were present in the riparian zone of the Weinan section of the Wei River (Table 2), and we investigated the effects of Fe(II) and DOC on the process of natural attenuation of NO_3_^−^-N in the riparian zone through microcosmic incubation experiments.

**Figure 6:**
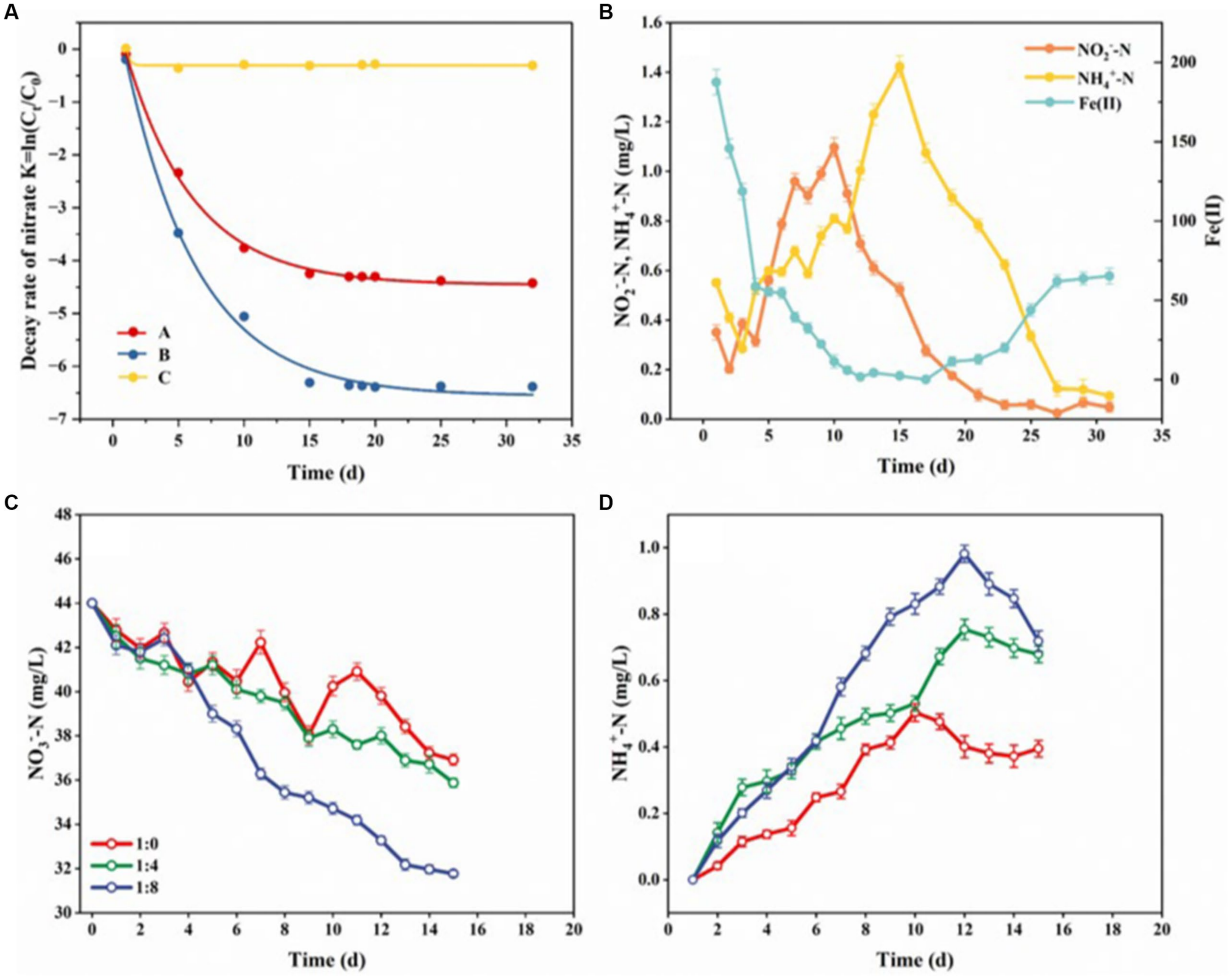
Patterns of NO_3_^−^-N decay rates and material changes in sediments in riparian zones under different exogenous conditions. **(A,B)** Show the effect of Fe(II) on sediment NO_3_^−^-N decay rates and the content of NO_3_^−^-N, Fe(II), NO_2_^−^-N, and NH_4_^+^-N. **(C,D)** Show the effect of DOC on NO_3_^−^-N and NH_4_^+^-N in sediments. C_t_ is the content of NO_3_^−^-N at t (d), C_0_ is the initial content of NO_3_^−^-N, **(A)** is the *in situ* riparian zone sediments, **(B)** is the sediment added with Fe(II), **(C)** is the sediment added with Fe(II) and sterilized, 1:0 indicates a DOC: NO_3_^−^ of 0:1 sediment, 1:4 indicates a DOC: NO_3_^−^ of 4:1 sediment, and 1:8 1:0 indicates a DOC: NO_3_^−^ of 8:1 sediment. 1, Error bars represent standard deviations.

We observed that compared with the *in situ* riparian zone sediments and the sediments supplemented with Fe(II) combined with sterilization, the riparian zone sediments supplemented with Fe(II) only had higher NO_3_^−^-N reduction rates (Figure 6A), which proved that there may be a microbial-driven nitrate-dependent ferrous oxidation process (NDFO) in the riparian zone, which can reduce NO_3_^−^ to NO_2_^−^, N_2_O, and N_2_. Additionally, Supplementary Figure S3 showed that the riparian zone sediment consumed a large amount of DOC in the system after the addition of Fe(II), which further indicated that the microorganisms involved in the NDFO process were mainly chemoenergetic, inorganic allotrophic or parthenogenic and used DOC as a carbon source. In the first 10 days of incubation, the contents of NO_3_^−^-N and Fe(II) in the system showed a sharp decline, and the NO_3_^−^-N was completely consumed to the minimum (0.07 mg/L, Figure 6B and Supplementary Figure S4), while the contents of NO_2_^−^-N and NH_4_^+^-N showed a significant upward trend (Figure 6B). This result indicated that the NDFO reaction (Fe(II) + NO_3_^−^ → Fe(III) + NO_2_^−^) was carried out in the riparian zone at this stage, which may be accompanied by denitrification (NO_3_^−^ → NO_2_^−^), nitrogen mineralization (DON → NH_4_^+^) and DNRA reaction (NO_3_- → NH_4_^+^). During the 11–30 days of culture, the content of NO_3_^−^-N in the sediments remained at the minimum value, and the content of NO_2_^−^-N showed a significant downward trend, while the content of NH_4_^+^-N increased first and then decreased, and the content of Fe(II) decreased first and gradually increased after 20 days (Figure 6B and Supplementary Figure S4), indicating that NDFO (Fe(II) + NO_2_^−^ → Fe (III) + N_2_), iron ammonia oxidation (Feammox, Fe(III) + NH_4_^+^ → Fe(II) + N_2_), and anammox (NO_2_^−^ + NH_4_^+^ → N_2_) may exist at this stage. Thus, the Fe-N cycle exists in the riparian zone sediments and Fe(II) accelerates the attenuation of NO_3_^−^-N in the riparian zone sediments through the NDFO reaction driven by microorganisms, while the coupling of Feammox with the NDFO process achieved the recycling of Fe within the system.

We also observed that under different carbon-nitrogen ratios (DOC: NO_3_^−^), the content of NO_3_^−^-N in the reaction system showed a downward trend (Figure 6C), while the content of NH_4_^+^ -N showed a trend of rapid increase and then gradual decrease (Figure 6D), and at the same time, the higher the value of DOC: NO_3_^−^, the more dramatic the changes in the NO_3_^−^-N and NH_4_^+^-N content (Figures 6C,D). In the pre-culture period (first 10 d), the NO_3_^−^-N content in the system decreased and the NH_4_^+^-N content increased, which might be attributed to the promotion of DNRA reaction by high carbon-nitrogen ratios. Numerous reports have shown that DOC is the substrate for DNRA and denitrification processes, denitrification process is a competitive advantage when the carbon-nitrogen ratios in the soil is lower than 2.5, but DNRA reaction occurs preferentially when the value of carbon-nitrogen ratios is higher than 4, and the sufficient DOC is beneficial to promote the activity and growth of DNRA bacteria (Tiedje et al., 1983; Wang et al., 2020a; Chen et al., 2022). In the later stage of culture (after 10 d), the content of NO_3_^−^-N and NH_4_^+^-N in the system decreased gradually, which may be due to the anammox. At this stage, DOC and NO_3_^−^-N in the system were consumed by denitrification and DNRA reaction, resulting in NO_3_^−^-N and NH_4_^+^-N, which provided sufficient substrate for anammox reaction and promoted the growth of anammox bacteria. Hence, in the riparian zone of Weinan section of Wei River, sufficient DOC increases the carbon-nitrogen ratios in the environment, which promotes the DNRA reaction and accelerates the attenuation of NO_3_^−^-N.

### 3.5. Correlation between environmental parameters, nitrogen transformation gene abundance and nitrogen removal rate

As the riparian zone is an active biogeochemical region with naturally developed physicochemical and microbiological gradients that are key drivers of the nitrogen cycle (Hou et al., 2017). We observed that D_total_ was significantly positively correlated with A_total_ and negatively correlated with R_DNRA_ (Supplementary Figures S5, S6), which suggested that denitrification and anammox process in the riparian zone had a mutually reinforcing relationship. Denitrification could provide electron acceptors (NO_2_^−^-N) for the anammox process, and the consumption of organic carbon by denitrifying bacteria could alleviate its inhibitory effect on anammox bacteria (Hou et al., 2015; Liu et al., 2019). On the other hand, denitrification and DNRA showed a competitive relationship, competing for the common electron donor DOC and electron acceptor NO_3_^−^ in environmental media. Therefore, the synergistic and competitive relationship among denitrification, anammox and DNRA jointly promoted the nitrogen removal process in the riparian zone.

**Figure 7:**
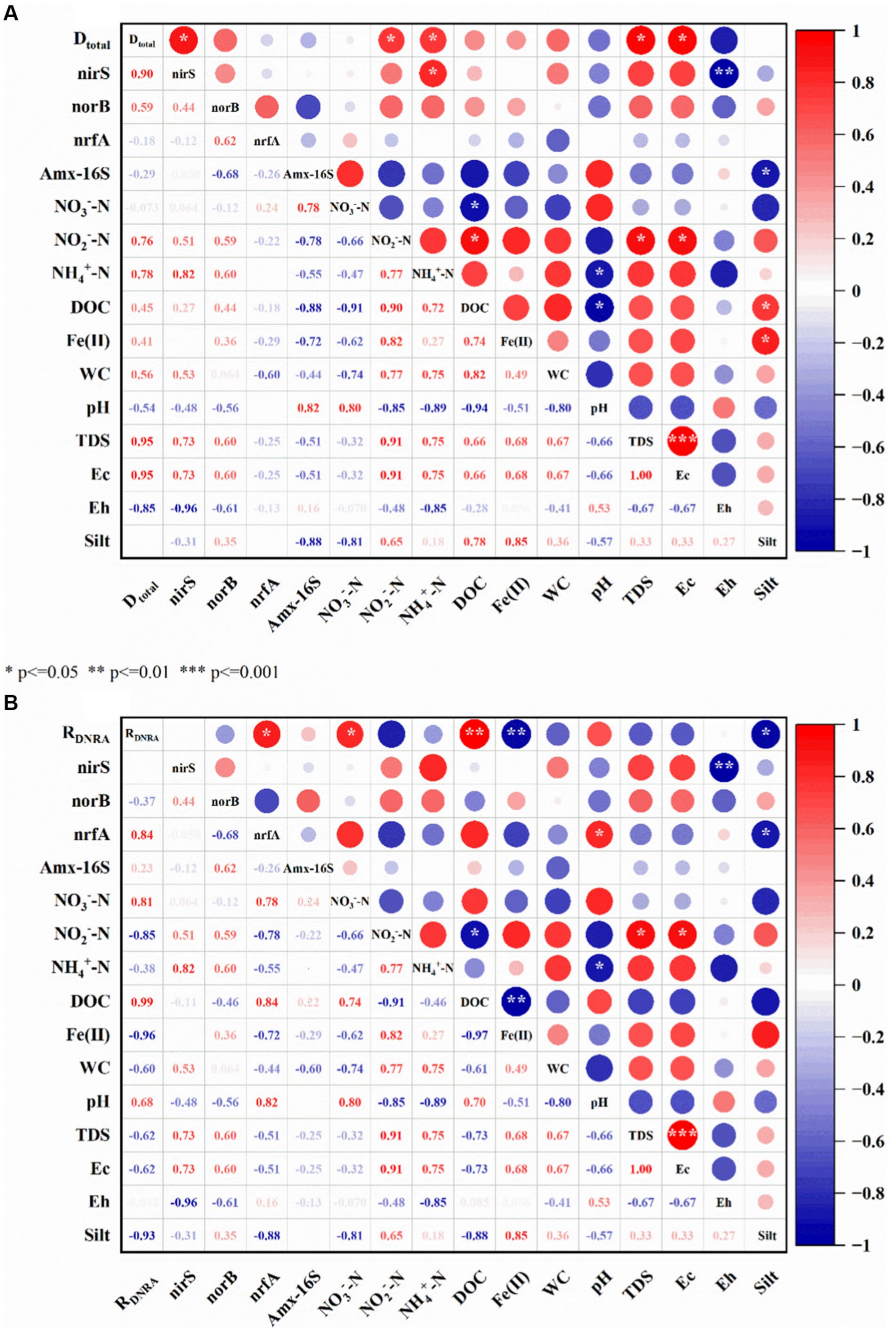
Analysis of driving factors of nitrogen removal rate. WC: water content, red represents a positive correlation, blue represents a negative correlation, the deeper the color, the stronger the correlation, the size of the circle is the correlation coefficient, *, **, *** represents the significant statistics at *p* < 0.05, 0.01, and 0.001, **(A)** is the driving factor affecting the denitrification rate, **(B)** is the driving factor affecting the DNRA rate.

In the riparian zone of Weinan section of Wei River, DNRA and denitrification are the most active in the attenuation process of nitrate. For the denitrification process, TDS, EC, NH_4_^+^ and NO_2_^−^ were the main environmental factors affecting D_total_, which were significantly positively correlated with D_total_ (*p* < 0.05, Figure 7A). This conclusion was consistent with the results of Magalhaes et al. (2005) in the intertidal sediments of Douro estuary in Portugal and Liu et al. (2018) in the sediments of Yangtze River lakes, these substances directly or indirectly provide electron acceptor/donor or energy for denitrifying bacteria to promote denitrification. A large number of studies have shown that nitrate is the reaction substrate of denitrification and is proportional to the denitrification rate (Mulder et al., 1995; Yao et al., 2018; Broman et al., 2021). Interestingly, the correlation between NO_3_^−^ content and D_total_ in this study was not strong and negatively correlated (Figure 7A), which may be related to the NO_3_^−^ content and the abundance of denitrifying bacteria in the riparian zone. The electron affinity of NO_3_^−^-N and NO_2_^−^-N was different, and there was a competitive electron relationship (Xing et al., 2016). In the W1, W3, and W5 layers containing sludge, the contents of DOC, NH_4_^+^-N and NO_2_^−^-N were significantly higher (Table 2 and Figure 2A). Therefore, the electrons provided by a large amount of DOC may preferentially flow to NO_2_^−^-N for denitrification (nitrite reduction) or DNRA reaction. While in the W2 and W4 layers with higher NO_3_^−^-N content, the DOC content and denitrification function (*nirS, norB*) microbial abundance were lower (Table 2 and Figures 4A,B), and the denitrification process might be inhibited. Accordingly, D_total_ in the riparian zone was significantly positively correlated with DOC and NO_2_^−^ contents, but not with NO_3_^−^ contents in sediments.

For the DNRA process, NO_3_^−^, sediment lithology (Silt), DOC, Fe(II) and NO_2_^−^ were the main environmental factors affecting R_DNRA_ (*p* < 0.05, Figure 7B). Among them, NO_3_^−^ and Fe(II) were significantly positively correlated with R_DNRA_, and the other environmental factors were significantly negatively correlated with R_DNRA_. Fetzer and Li et al. found that the microbial community of riparian sediments is very sensitive to lithologic structure, coarse particles promote microbial activity and accelerate material transport, and fine particles will block exchange channels, reduce microbial activity and hinder solute migration and transformation (Fetzer et al., 2017; Wang et al., 2020b). The lithology of the sediments in the riparian zone of the Weinan section of the Wei River was dominated by silt, followed by fine sand, and these fine particles might inhibit the DNRA reaction. Microcosmic experiments showed that there was a coupled Fe-N cycle in the riparian zone, and Fe(II) would promote the attenuation of NO_3_^−^-N through NDFO action, which reduced the reaction substrate of DNRA and inhibited the DNRA reaction. In contrast, NO_3_^−^ and DOC provided electron acceptors and electron donors, respectively, for the DNRA reaction (Li Z et al., 2020; Chang et al., 2021), which facilitated the DNRA reaction.

In addition, D_total_ was significantly and positively correlated with the abundance of denitrification functional genes *nirS* and *nroB*, and R_DNRA_ was also significantly and positively correlated with the abundance of DNRA functional gene *nrfA*. This conclusion is consistent with the research results of China’s Yangtze River estuary (Zheng et al., 2021), Northeast China Sea (Chang et al., 2021), and high-altitude rivers (Zhang et al., 2020). This further proves that the nitrogen removal process of riparian sediments is mediated by the redox reaction of different microorganisms. In addition, in the study of soil nitrogen cycle, it was found that the microorganisms involved in denitrification and DNRA processes usually lived in the same environment, and the optimal pH range was relatively close, denitrification was 6–8, and DNRA was 5–9. High pH will promote nitrate reduction through DNRA reaction and weaken the intensity of denitrification (Stevens et al., 1998; Fernandes et al., 2012). Interestingly, in this study, the pH of the sediment environment was 7.77–8.02, and the correlation analysis showed that pH was negatively correlated with D_total_ and denitrifying bacteria abundance, and significantly positively correlated with R_DNRA_ and DNRA bacteria abundance (*p* < 0.05, Figure 7). It indicated that the pH of the sedimentary environment in the riparian zone was more suitable for the growth of DNRA bacteria.

In general, compared with the permanent submerged sediments, the riparian sediments in the Weinan section of the Wei River are in a dry-wet alternation state. The interaction between river water and groundwater causes the fluctuation of water level in the riparian zone, which changes the physical and chemical properties of the sediments, and then affects the microbial community structure and the distribution characteristics of inorganic nitrogen. The abundance of nitrogen-converting microorganisms, NO_X_^−^, Fe(II) and DOC jointly drove the N-loss process in the riparian zone.

## 4. Conclusion

This study investigated N-loss processes and the diversity and abundance of associated microbial communities in the heterogeneous riparian zone of the Weinan section of the Wei River. The results showed that the combined effects of denitrification (17.76–35.19 μmol·N·kg^−1^·h^−1^), DNRA (9.37–12.47 μmol·N·kg^−1^·h^−1^) and anammox (4.14–5.77 μmol·N·kg^−1^·h^−1^) achieved the natural attenuation of nitrogen in the riparian zone, and denitrification (accounting for 51.6–69.9%) was the dominant process. In addition, the riparian zone sediments harbor diverse and abundant microbial communities that mediate these processes. The abundance of functional genes and environmental factors (especially DOC and Fe(II)) are also the constraints of sediment nitrogen removal rate in riparian zone. These results suggest that riverbed sediments are a hot area for nitrogen removal in riparian zones, which is of great significance for strengthening the control of river nitrogen pollution. This study has important implications for understanding and managing the water quality and ecosystem health of inland rivers in arid and semi-arid regions.

## Data availability statement

The datasets presented in this study can be found in online repositories. The names of the repository/repositories and accession number(s) can be found below: NCBI, PRJNA1002244.

## Author contributions

YW, FYe, RL, FYa, and FG performed the material preparation, data collection, and analysis. FYe, YS, and LD wrote the first draft of the manuscript. FYe, YX, and YS wrote the revised manuscript. All authors contributed to the conception and design of the study, commented on previous versions of the manuscript, read, and approved the final manuscript.

## Funding

This study was funded by the National Natural Science Foundation of China (Nos. 41877190 and 42277061), the Key Research and Development Program of Shaanxi (Nos. 2020ZDLSF06-04 and 2021ZDLSF05-05).

## Conflict of interest

FYa, RL, and FG were employed by Power China Northwest Engineering Corporation Limited.

The remaining authors declare that the research was conducted in the absence of any commercial or financial relationships that could be construed as a potential conflict of interest.

## Publisher’s note

All claims expressed in this article are solely those of the authors and do not necessarily represent those of their affiliated organizations, or those of the publisher, the editors and the reviewers. Any product that may be evaluated in this article, or claim that may be made by its manufacturer, is not guaranteed or endorsed by the publisher.
