# Supplementary material for: Nitrogen removal in freshwater sediments of riparian zone: N-loss pathways and environmental controls
Source: Front Microbiol. 2023 Aug 17;14:1239055. doi: 10.3389/fmicb.2023.1239055 (PMC10469909; doi:10.3389/fmicb.2023.1239055)
Supplement: Supplementary file 1 [file Data_Sheet_1.docx]

**Supplementary Information**

**Nitrogen removal in freshwater sediments of riparian zone: N-loss pathways and environmental controls**

Fei Ye^1,2^, Lei Duan^1,2^, Yaqiao Sun^1,2*^, Fan Yang ^3,4^, Rui Liu^3,4^, Fan Gao^3,4^, Yike Wang^1,2^, Yirong Xu^1,2^

^1^ School of Water and Environment, Chang’an University, Xi’an 710054, China;

^2^ Key Laboratory of Subsurface Hydrology and Ecological Effects

in Arid Region, Ministry of Education, Chang'an University,

P.R. China;

^3^ Power China Northwest Engineering Corporation Limited, Xi’an, Shaanxi 710065, China.

^4.^ Shaanxi Union Research Center of University and Enterprise for River and Lake Ecosystems Protection and Restoration, Xi’an, Shaanxi 710065, China.

* Corresponding author.

E-mail address: sunyaqiao@126.com (Yaqiao Sun)

**Supplementary Tab. S1** Reaction systems for investigating the effect of Fe(Ⅱ) on nitrogen conversion processes

| Sample | Number | NaNO_3_ | FeSO_4_·7H_2_O | Microorganisms |
| --- | --- | --- | --- | --- |
| W | A | 44mg/L | / | / |
|  | B | 44mg/L | 7mmol/L | / |
|  | C | 44mg/L | 7mmol/L | Sterilisation |

Note: The sediment samples were sterilised using an autoclave (OMY SX-500, Japan) at 125°C, a sterilisation pressure >0.1Mpa and a sterilisation time of 120min.

**Supplementary Tab. S2** Reaction systems for investigating the effect of DOC on nitrogen conversion processes

| Sample | Number | NaNO_3_ | CH_3_COONa | C/N |
| --- | --- | --- | --- | --- |
| W | D | 44mg/L | / | 0:1 |
|  | E | 44mg/L | 176mmol/L | 4:1 |
|  | F | 44mg/L | 352mmol/L | 8:1 |


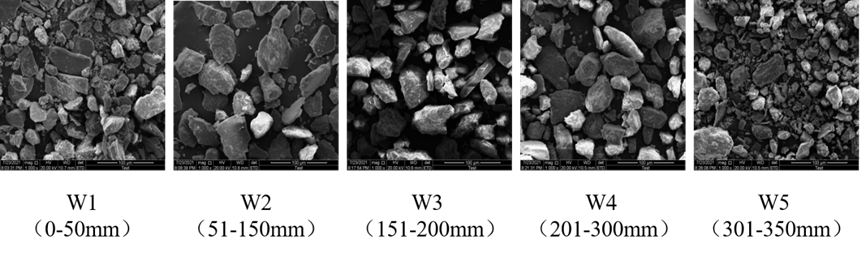


**Supplementary Fig. S1** SEM electron microscope scans of sediment samples from the riparian zone of the Wei River

**Supplementary Tab. S3** The α-diversity indices of anammox bacterial and denitrifying communities.

|  |  | W1 | W2 | W3 | W4 | W5 |
| --- | --- | --- | --- | --- | --- | --- |
| nirS | Shannon | 8.69 | 8.46 | 8.65 | 7.51 | 8.42 |
|  | Simpson | 0.99 | 0.99 | 0.99 | 0.99 | 0.99 |
|  | Ace | 1335.40 | 1061.70 | 1735.10 | 749.30 | 1053.10 |
|  | Chao1 | 1469.67 | 1291.46 | 1904.14 | 1090.55 | 1236.12 |
|  | Coverage | 99.79% | 99.73% | 99.68% | 99.67% | 99.79% |
| norB | Shannon | 6.10 | 4.54 | 5.38 | 3.90 | 2.53 |
|  | Simpson | 0.96 | 0.86 | 0.91 | 0.77 | 0.69 |
|  | Ace | 538.00 | 382.90 | 724.40 | 355.30 | 197.80 |
|  | Chao1 | 589.92 | 517.11 | 811.15 | 377.94 | 256.01 |
|  | Coverage | 99.81% | 99.77% | 99.71% | 99.89% | 99.89% |
| 16S | Shannon | 1.64 | 3.42 | 1.67 | 3.58 | 1.26 |
|  | Simpson | 0.58 | 0.85 | 0.50 | 0.87 | 0.52 |
|  | Ace | 48.80 | 179.40 | 100.90 | 300.50 | 98.80 |
|  | Chao1 | 63.55 | 215.79 | 129.47 | 385.53 | 132.27 |
|  | Coverage | 99.97% | 99.89% | 99.93% | 99.78% | 99.91% |
| nrfA | Shannon | 7.42 | 8.34 | 8.29 | 7.15 | 8.11 |
|  | Simpson | 0.98 | 0.99 | 0.99 | 0.99 | 0.99 |
|  | Ace | 1317.70 | 1870.80 | 2016.90 | 778.60 | 1409.00 |
|  | Chao1 | 1588.18 | 2179.62 | 2226.82 | 921.53 | 1608.88 |
|  | Coverage | 99.68% | 99.58% | 99.62% | 99.82% | 99.71% |

Note: Microbial alpha diversity (e.g., ACE, Chao1, Shannon and Simpson) was estimated based on 90% OTUs clusters



 **Supplementary Fig. S2** N_2_O content of sediments at different depths in the riparian zone of the Weinan section of the Weihe River.





**Supplementary Fig. S3** Beginning and end values of DOC content in the system under different exogenous conditions (Red indicates the initial value of DOC, blue and yellow indicate the DOC content at the end of the incubation, B represents the addition of Fe(II), C represents the addition of Fe(II) and inactivation of microorganisms).





**Supplementary Fig. S4** NO_3_^-^-N content of riparian zone sediments under different exogenous conditions (A indicates in situ riparian zone sediments, B indicates Fe(II) added sediments, C indicates Fe(II) added and sterilized treated sediments)


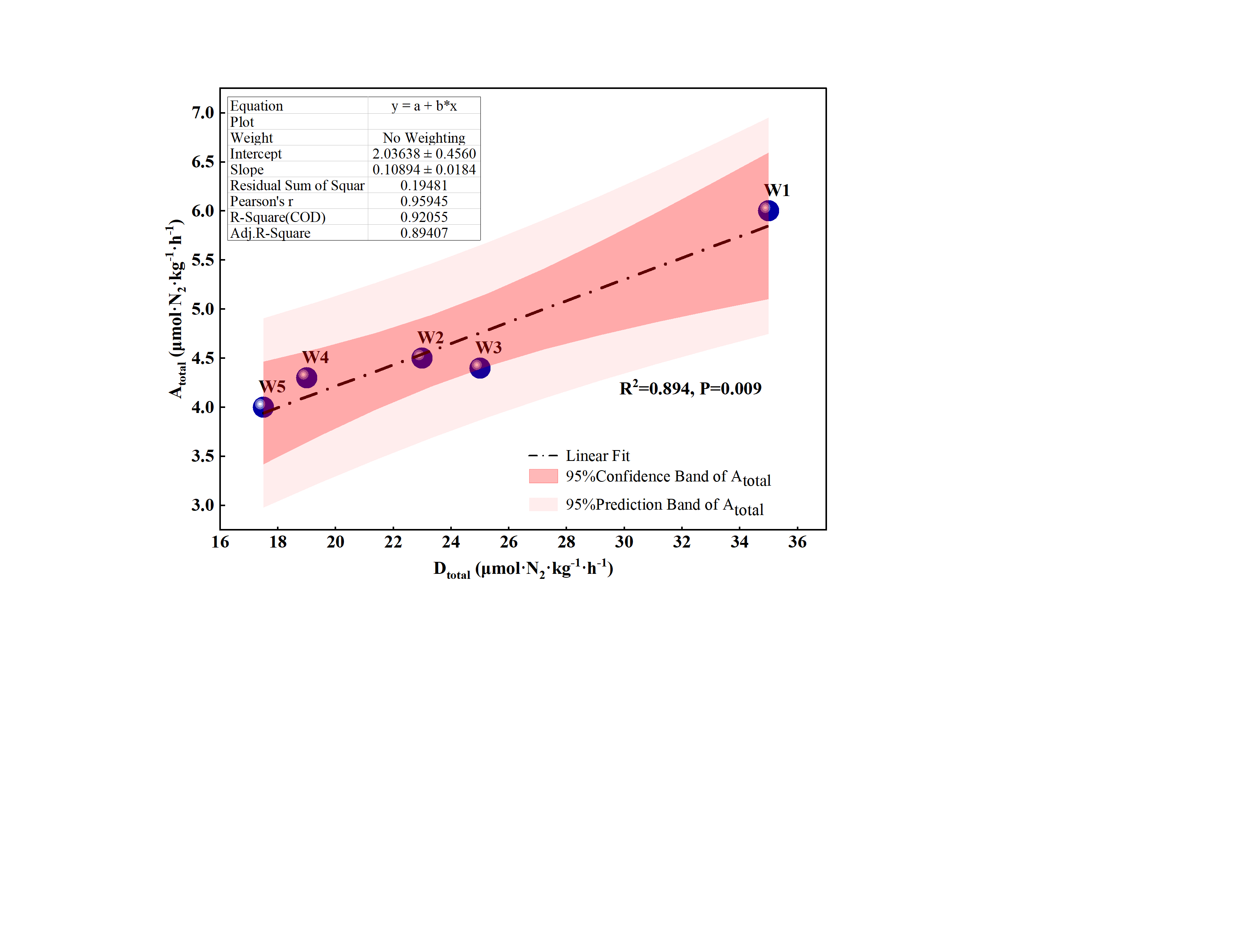


**Supplementary Fig. S5** Relationship between denitrification rate and anaerobic ammonia oxidation rate.





**Supplementary Fig. S6** Relationship between denitrification rate and DNRA rate
